# Time Course of Detection of Human Male DNA from Stained Blood Sample on Various Surfaces by Loop Mediated Isothermal Amplification and Polymerase Chain Reaction 

**DOI:** 10.1155/2018/2981862

**Published:** 2018-03-22

**Authors:** Panan Kanchanaphum

**Affiliations:** Department of Medical Science, Faculty of Science, Rangsit University, Pathum Thani, Thailand

## Abstract

This study explores determining the sex of humans from blood stains taken from different surfaces and compares the time course of detection with the conventional PCR, Conventional Loop Mediated Isothermal Amplification (LAMP), and LAMP-Lateral Flow Dipstick (LFD). For the DNA templates, 7 male and 7 female blood stained samples were extracted and added to LAMP and PCR reaction solution to amplify the SRY gene. The DNA samples were extracted from the following blood stained materials: cloth, wood, clay, and tile. Then, the samples were stored at room temperature for 1, 7, 30, and 60 day(s). After the DNA amplification, the gel electrophoresis process was applied to detect LAMP product. The LFD was combined with the LAMP to detect LAMP product on the male cloth samples. For the male samples, the time course of detection on the first and seventh days indicated positive for both LAMP and PCR products on all the surfaces while no DNA amplification was found on any of the female samples. On day 30, positive LAMP product was still found on all the male samples. However, it had faded on the tiles. Moreover, all the male samples, which had tested positive for PCR product, were blurred and unclear. On day 60, LAMP product was still found on all the male samples. Conversely, the PCR method resulted in no bands showing for any of the male samples. However, the LAMP-LFD method detected product on all the male samples of cloth. The results show that the LAMP is an effective, practical, and reliable molecular-biological method. Moreover, the LFD can increase the efficiency and sensitivity of the LAMP, making it more suitable for field studies because gel electrophoresis apparatus is not required.

## 1. Introduction

Biological evidence can be used during a criminal investigation to determine the sex of victims [1]. Ordinarily, the anatomical characteristics of external genitalia, such as the gonads, ovaries, or testes, are used to determine the sex of an unidentified body. Currently, the molecular-biological method of identifying and determining human sex depends on the Polymerase Chain Reaction (PCR) method.

Loop Mediated Isothermal Amplification (LAMP) is a relatively new, less complicated, more rapid DNA detection method, which has been developed as an alternative to PCR. Moreover, LAMP has been used to determine the sex of bovine embryos, female calves, and* Columbidae* birds [2, 3] as well as detect viruses such as Taura syndrome in shrimp [4]. Kanchanaphum et al. [5] developed and used the LAMP method to determine the sex of humans using the SRY gene as a target.

The LAMP method has been used to amplify DNA targets under isothermal conditions in the temperature range of 60°C–65°C for 45–60 minutes [6]. At least two sets of primer (inner primer and outer primer) used in LAMP were specific at six different regions located within the target sequence and primary DNA amplification began by the inner primer set. The characteristic intermediary DNA structure formed by LAMP, called a stem-loop DNA fragment, was generated and large amounts of DNA products were produced by an autocycle reaction [3].

As a replacement for conventional LAMP, which requires product detection by gel electrophoresis, a simplified, less time-consuming field-based detection system using a chromatographic lateral flow dipstick (LFD) has been developed for applications such as the detection of shrimp pathogens, that is, white spot syndrome virus (WSSV) [7], Penaeus monodon nucleopolyhedrovirus (PemoNPV) [8], and the infectious hypodermal and hematopoietic necrosis virus (IHHNV) [9]. In this case, a generic LFD strip (Milenia GenLine HybriDetect, Germany) was used to detect biotin-labeled LAMP products that were hybridized with a FITC-labeled DNA probe conjugated with a gold-labeled anti-FITC antibody. The combination of LAMP and LFD (LAMP-LFD) required 5–10 min as compared to 45–60 min for gel electrophoresis. The LFD method does not require complicated equipment; users dip the LFD strip into an LFD-buffer and determine the results by naked eye.

The sex-determining region Y or SRY gene on the Y chromosome has been used for sex determination [10, 11]. The SRY gene translates to a protein with a central high mobility group domain (HMG) of about 78 amino acids [12]; this stimulates the undifferentiated gonad, which develops into a testis. Sex determination using PCR to amplify the SRY gene has also been reported [10, 11].

Therefore, the objective of this study is to determine the sex of humans from the blood stained onto different surfaces and compare the time course of detection with the conventional PCR, conventional LAMP, and LAMP-LFD methods.

## 2. Materials and Methods

### 2.1. DNA Template Preparation

The DNA templates used in this research were kindly supplied by Dr. Ratchanok Kumsiri. Initially, the DNA template for the bloodstained samples was prepared by spreading a few drops of blood on 4 different materials: cloth, wood plank, and clay. Then, the samples were dried and stored at room temperature. Next, a cotton bud soaked in distilled water was swabbed onto the surface of the material to collect the blood stains. After that, the cotton bud was immersed in a tube, which contained distilled water. Finally, the genomic DNA was extracted from the solution using the GF-1 Blood DNA extraction kit (Vivantis, Malaysia).

For the time of course detection, bloodstained samples taken from seven males and seven females were spotted on 4 different surfaces and then stored for 0, 1, 7, 30, and 60 days at room temperature. Next, the DNA was extracted from the bloodstained samples, as described above. Subsequently, these DNA solutions were used as a template for LAMP and PCR amplification.

### 2.2. PCR Reaction and Analysis

The PCR amplification reaction contained 1x* Taq* DNA polymerase buffer, 400 mM betaine, 1.2 mM dNTPs, 0.8 *μ*M F3 and B3 primers, and 8 U* Taq *DNA polymerase (New England Biolabs) as well as 5 ng of each DNA extract as a template in a final volume of 25 *μ*l. The cycling conditions included a single initial denaturation at 94°C for 2 min followed by 35 cycles at 90°C for 30 sec (denaturation), 60°C for 30 sec (annealing), and 72°C for 30 sec (extension) and a final extension step at 72°C for 5 min. After the PCR amplification, the PCR products were analyzed by electrophoresis using 1.5% agarose gel stained with ethidium bromide. The gels were visualized under ultraviolet light.

### 2.3. LAMP Reaction and Analysis

The SRY primers used in this research were designed based on the human SRY gene (GenBank accession number JQ811934), as described in [6]. The reaction mixture contained 1x* Bst *DNA polymerase buffer, 5 mM MgSO_4_, 400 mM betaine, 1.2 mM dNTPs, 0.8 *μ*M F3 and B3 primers, 2 *μ*M FIP and BIP primers, and 8 U* Bst *DNA polymerase (New England Biolabs), as well as 5 ng of each DNA extract as a template in a final volume of 25 *μ*l. The reaction was carried out at 65°C for 45 min and was followed by inactivation of the enzyme at 80°C for 5 min, as described by Whitfield et al. [13]. The LAMP products were analyzed by loading 10 *μ*l of LAMP product on 1.5% agarose gel. After the gel electrophoresis, the gel was stained with ethidium bromide and visualized under ultraviolet light.

### 2.4. Biotin-Labeling LAMP Condition

All the cloth samples taken from males over the time period (0–60 days) were analyzed using the LAMP-LFD method. The inner primer FIP was labeled with a biotin group at the 5′ end. The reaction mixture contained 1x* Bst *DNA polymerase buffer, 5 mM MgSO_4_, 400 mM betaine, 1.2 mM dNTPs, 0.8 *μ*M F3 and B3 primers, 2 *μ*M FIP and BIP primers, and 8 U* Bst *DNA polymerase (New England Biolabs) as well as 5 ng of each DNA extract as a template in a final volume of 25 *μ*l. The reaction was carried out at 65°C for 45 min and was followed by inactivation of the enzyme at 80°C for 5 min.

### 2.5. Lateral Flow Dipstick (LFD) Assay

**Figure 1:**
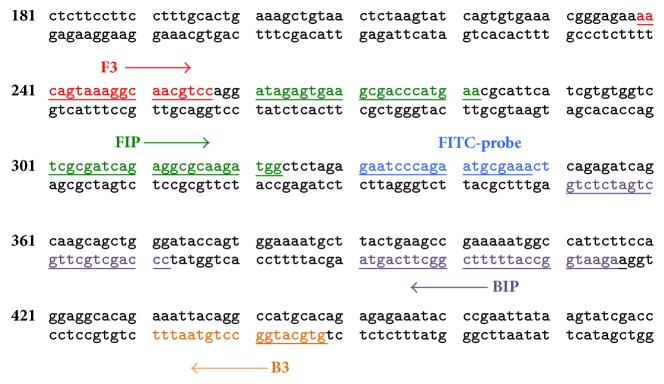
Nucleotide sequence of human SRY gene (GenBank accession number JQ811934). Schematic diagram of two inner (FIP and BIP) and two outer (F3 and B3) primer for LAMP are shown by arrows. The FITC-labeled probe sequence is shown under word “FITC-probe.”

A DNA probe was designed from the SRY sequence between the FIP and BIP regions (Figure 1). The DNA probe was labeled with FITC at the 5′ end and synthesized by Pacific Science, Thailand. Twenty pmole of DNA probe was added to the biotin-labeled primer LAMP reaction without enzyme inactivation, as recommended in [14]. After hybridization at 65°C for 5 min, 8 *μ*l of the hybridized product was added to 150 *μ*l of assay buffer in a new tube. Then, an LFD strip was dipped into the mixture. The results appeared within 10 min.

## 3. Results

**Figure 2:**
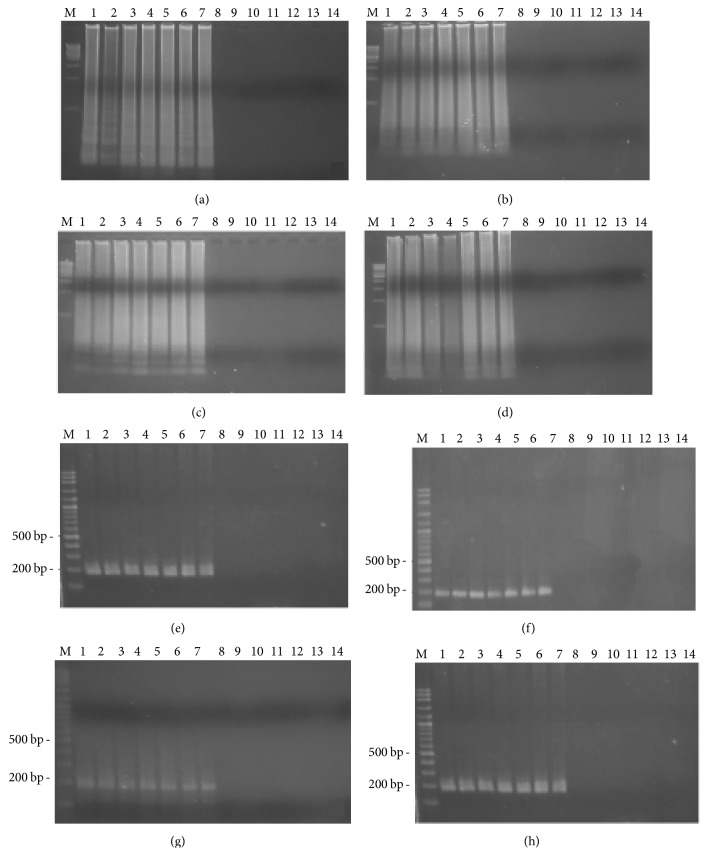
Time course detection of SRY locus by LAMP and PCR from 7 males' DNA and 7 females' DNA samples stained on different surface ((a) = cloth, (b) = wood plank, (c) = clay, and (d) = tile) in LAMP and ((e) = cloth, (f) = wood plank, (g) = clay, and (h) = tile) in PCR for the first day. Lane M = 1 kb DNA ladder in LAMP and 100 bp DNA ladder in PCR. Lane 1–7 = male numbers 1–7. Lane 8–14 = female numbers 1–7.

**Figure 3:**
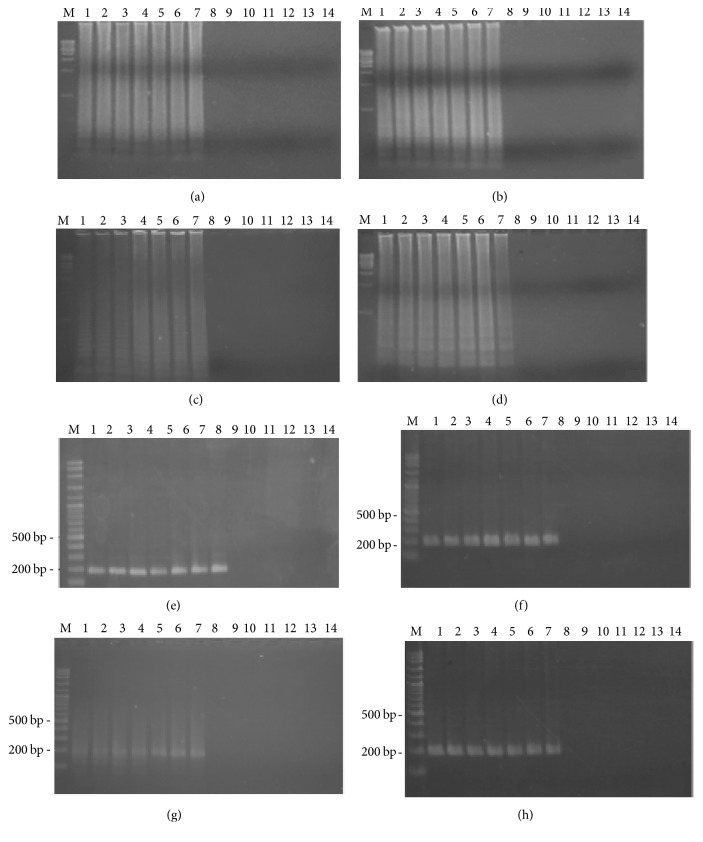
Time course detection of SRY locus by LAMP and PCR from 7 males' DNA and 7 females' DNA samples stained on different surface ((a) = cloth, (b) = wood plank, (c) = clay, and (d) = tile) in LAMP and ((e) = cloth, (f) = wood plank, (g) = clay, and (h) = tile) in PCR for the seventh day. Lane M = 1 kb DNA ladder in LAMP and 100 bp DNA ladder in PCR. Lane 1–7 = male numbers 1–7. Lane 8–14 = female numbers 1–7.

**Figure 4:**
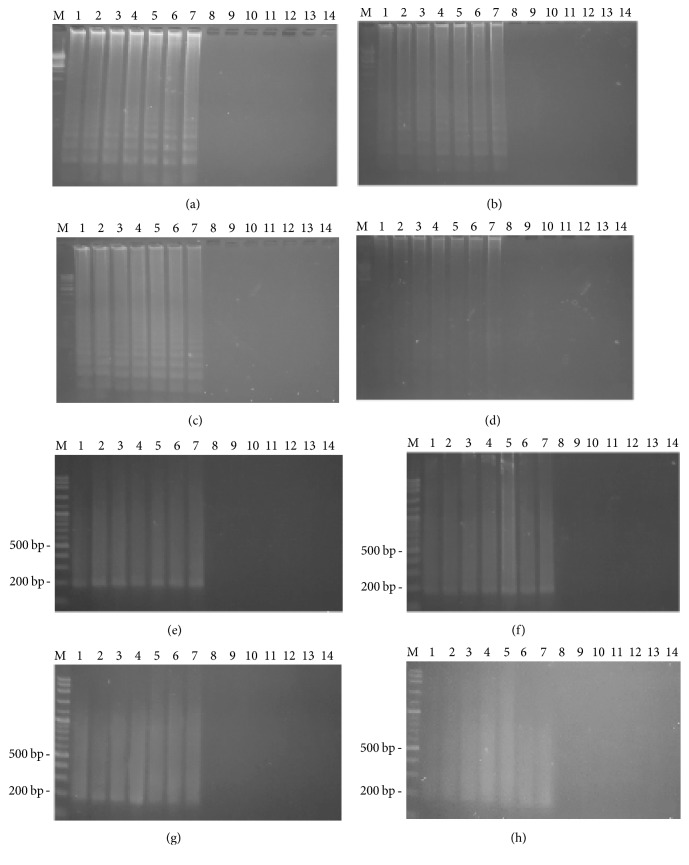
Time course detection of SRY locus by LAMP and PCR from 7 males' DNA and 7 females' DNA samples stained on different surface ((a) = cloth, (b) = wood plank, (c) = clay, and (d) = tile) in LAMP and ((e) = cloth, (f) = wood plank, (g) = clay, and (h) = tile) in PCR for the thirtieth day. Lane M = 1 kb DNA ladder in LAMP and 100 bp DNA ladder in PCR. Lane 1–7 = male numbers 1–7. Lane 8–14 = female numbers 1–7.

**Figure 5:**
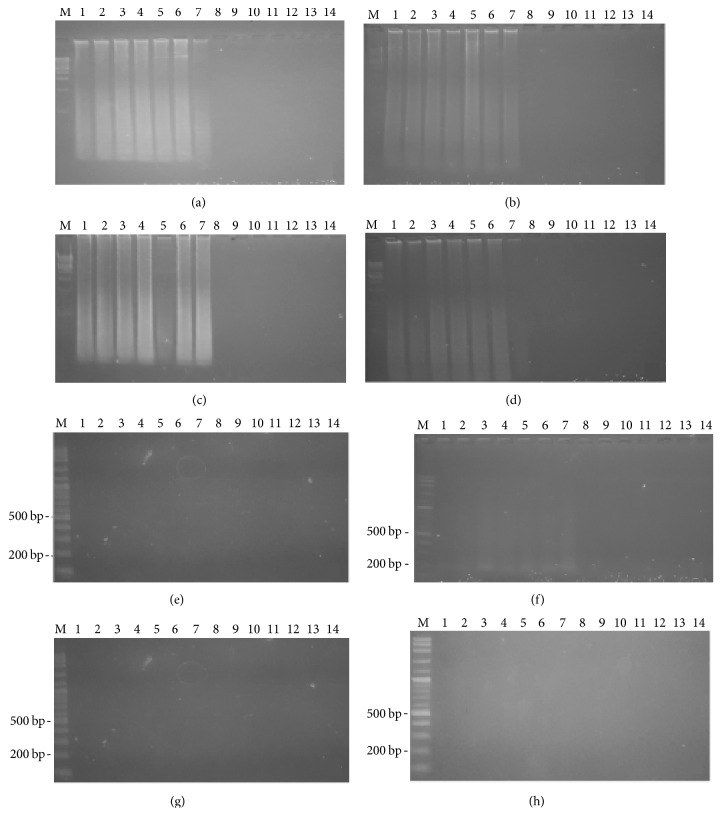
Time course detection of SRY locus by LAMP and PCR from 7 males' DNA and 7 females' DNA samples stained on different surface ((a) = cloth, (b) = wood plank, (c) = clay, and (d) = tile) in LAMP and ((e) = cloth, (f) = wood plank, (g) = clay, and (h) = tile) in PCR for the sixtieth day. Lane M = 1 kb DNA ladder in LAMP and 100 bp DNA ladder in PCR. Lane 1–7 = male numbers 1–7. Lane 8–14 = female numbers 1–7.

**Figure 6:**
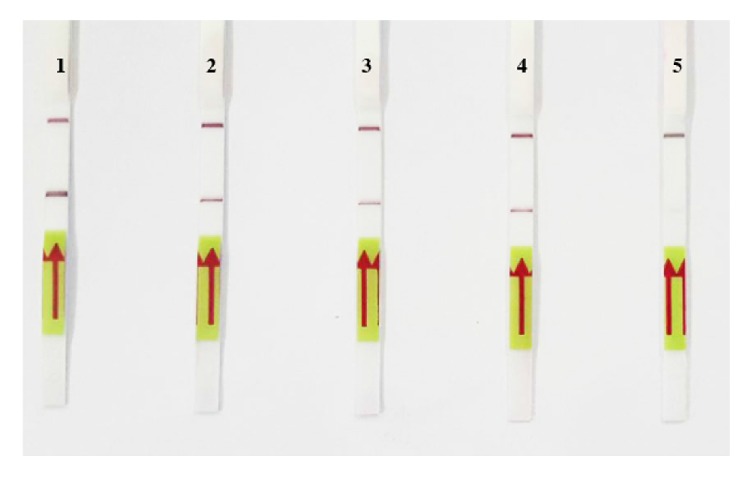
Time course of detection of SRY locus by LAMP-LFD from males' DNA sample stained on cloth. 1 = the first day, 2 = the seventh day, 3 = the thirtieth day, 4 = sixtieth day, and 5 = negative control.

Figure 2 shows the time course of detection for both the LAMP and PCR methods, for 4 different surfaces on the first day. For all the male samples, on 4 different surfaces, both the LAMP and PCR methods resulted in a positive band while no DNA amplification was found on any of the female samples. For the LAMP reaction, on the first day smeared bands were observed on the male samples. For the PCR reaction, 190 bp PCR product was detected on the male samples.  Figure 3 shows that, on day seven, both the LAMP and PCR methods resulted in a positive for the male samples. On day 30, LAMP product was still detected on the male samples. However, the positive band of LAMP observed on the tile samples had faded, as shown in Figure 4. Moreover, for all the male samples, on 4 different surfaces, the positive PCR product image had blurred, as shown in Figure 4. On day 60, for all the male samples, on 4 different surfaces, the LAMP method still resulted in a positive, as shown in Figure 5. Conversely, the PCR method resulted in no bands being visible for any of the male samples, as shown in Figure 5. However, the LAMP-LFD method detected product on all the male samples of cloth over (0–60) days while no amplification product was detected using the conventional LAMP method, as shown in Figure 6.

## 4. Discussion

Currently, in the field of molecular biology, the conventional PCR method is used by forensic scientists to determine human sex [15, 16]. However, this method requires sophisticated equipment and the procedure is time-consuming. To solve this problem, Notomi developed a novel LAMP method to identify the sex of humans, which does not require a thermal cycle machine [5]. Four primers based on 6 specific sequences on the target gene generate the ladder band. This method is fast and precise; the LAMP reaction time is only 1 hr because only one temperature (60–65°C) is necessary for DNA amplification. Another important advantage of the LAMP method is that the amplified product can be easily observed because white precipitation from magnesium pyrophosphate (Mg_2_P_2_O_7_) is visible to the naked eye [5] and as fluorescence in the presence of either ethidium bromide or PicoGreen during UV illumination.

In this research, the sensitivity of the LAMP method is compared to the PCR method in terms of the time course of detection. The results are in accordance with Kanchanaphum et al. who found the LAMP method to be more sensitive than the PCR method [5]. Analysis of the SRY gene revealed that product from the PCR method showed a smeared band on day 30 that disappeared by day 60 while LAMP gave positive results for the male samples until day 60. This demonstrates that the LAMP method does not require good quality DNA for the amplification. However, for the tiles, LAMP product on the male samples was not detected as thoroughly as it was on the other materials because the tiles had smooth, nonporous surfaces, which did not retain the DNA. Consequently, tiles were found to be the least suitable material for detecting DNA. The results of this research confirm that the LAMP method is more efficient than the conventional PCR method.

However, there are some techniques, which are able to increase the sensitivity of LAMP product detection that can replace gel electrophoresis such as the lateral flow dipstick method (LFD). This research found that the LFD method was a more useful and effective method because the results were clearer and easier to interpret than when gel electrophoresis was used. LFD is a simplified chromatographic field-based detection method, which has been developed for applications such as the detection of several shrimp pathogens, that is, white spot syndrome virus (WSSV) [7],* Penaeus monodon* nucleopolyhedrovirus (PemoNPV) [8], and infectious hypodermal and hematopoietic necrosis virus (IHHNV) [9].

## 5. Conclusion

The results of this research indicate that the LAMP method is more efficient, useful, and sensitive than the conventional PCR method of detecting DNA. The LAMP method in combination with LFD detection was found to be more sensitive than conventional PCR, which uses gel electrophoresis to detect the PCR product. The LAMP-LFD combination has some advantages over the PCR method: it does not require complicated equipment, the results of the DNA analysis are available relatively quickly, and it is portable enough to be carried around for field study.
